# Elimination of Young Erythrocytes from Blood Circulation and Altered Erythropoietic Patterns during Paraquat Induced Anemic Phase in Mice

**DOI:** 10.1371/journal.pone.0099364

**Published:** 2014-06-19

**Authors:** Nitin Bhardwaj, Rajiv K. Saxena

**Affiliations:** 1 School of Life Sciences, Jawaharlal Nehru University, New Delhi, India; 2 Faculty of Life Sciences and Biotechnology, South Asian University, New Delhi, India; National Institutes of Health, United States of America

## Abstract

Paraquat a widely used herbicide causes a variety of toxic effects on humans and animals. The present study is focused on the interaction of paraquat with the mouse erythroid system. Administration of paraquat (10 mg/kg body weight i.p. on alternate days in C57Bl/6 mice) induced a significant fall in blood erythrocyte count on 7, 14, and 21 day time points but the erythrocyte count reverted back to normal by 28^th^ day indicating the emergence of refractoriness to paraquat. A marked surge in the blood reticulocyte count was observed in paraquat treated mice that also subsided by 28^th^ day. Young erythrocytes in circulation were randomly eliminated from blood circulation in paraquat treated mice and a significant elevation in the level of reactive oxygen species (ROS) was also observed maximally the erythrocytes of this age group. Cells representing various stages of erythroid differentiation in bone marrow and spleen were identified and enumerated flow cytometrically based on their expression of Ter119 and transferrin (CD71) receptor. Proliferative activity of erythroid cells, their relative proportion as well as their absolute numbers fell significantly in bone marrow of paraquat treated mice but all these parameters were significantly elevated in spleens of paraquat treated mice. These changes were essentially restricted to the cells belonging to the two earliest stages of erythroid differentiation. Taken together our results indicate that paraquat treatment causes a transient anemia in mice resulting from random elimination of young circulating erythrocytes as well as depressed erythropoietic activity in bone marrow. Spleen erythropoietic activity however was elevated in paraquat treated mice.

## Introduction

Paraquat (N, N’-dimethyl-4,4′-bipyridinium dichloride) was first synthesized in 1880 but its action as a potent herbicide was discovered only in 1955 [1]. This compound soon became one of the most widely used herbicide in crop management. Paraquat kills plants rapidly by deactivating the photosynthetic mechanism. It also has considerable toxicity towards animals and humans and has widely been used for suicide [2]–[4]. Use of paraquat was banned in Europe in 2007 but the herbicide is still widely used in the rest of the world.

Ingestion of paraquat causes liver, lung, heart, and kidney failure within several days to several weeks [5]. Long-term exposures to paraquat causes lung and eye damage though reproductive/fertility damage was not found by the United States Environmental Protection Agency [6]. A link between the exposure to paraquat and Parkinson’s disease has also been reported [7]–[8]. Paraquat is a potent inducer of reactive oxygen species (ROS) and occurrence of anemia as a consequence of exposure to paraquat has also been documented [9]–[10]. In the present study we have developed a mouse model for paraquat induced anemia and have examined the changes in the turnover of erythrocytes of different age groups in blood circulation of paraquat treated mice. Attendant changes in erythropoietic activity in bone marrow and spleen were also examined.

Erythropoiesis is a multistep process that starts with the commitment of pluripotent hematopoietic stem cells (HSCs) progeny into erythroid line of differentiation [11]. Four distinct stages of erythroid differentiation have been identified on the basis of morphological features and membrane expression of transferrin receptors (CD71) and Ter119 molecules [12]–[14]. Bone marrow (BM) is the primary site of erythropoiesis that generates fresh erythrocytes to replace the aged erythrocytes eliminated from blood circulation. In anemia resulting from hematological disorder, blood loss or hypoxia, erythropoietic activity may be up-regulated as a compensatory mechanism and spreads to extra-medullary organs like spleen and liver [15]–[17].

Results of our present study show that the administration of paraquat results in an acute yet transient anemia in mice. Young erythrocytes that are not eliminated from circulation in control mice are eliminated at a significant rate in paraquat treated mice. Depressed proliferative activities and elevated cell death was observed in cells of erythroid lineage in the bone marrow of paraquat treated mice. In contrast, spleen erythropoietic activity was found to be significantly enhanced after paraquat administration suggesting that spleen may play a significant role in enhanced compensatory erythropoietic activity in paraquat treated mice.

## Materials and Methods

### Animals

Inbred C57BL/6 **male** mice (8–12 weeks old, 20–25 g body weight) were used throughout this study. Animals were bred and maintained in microbes free environment in the animal house facility at Jawaharlal Nehru University (JNU), New Delhi or obtained from the National Institute of Nutrition, Hyderabad. The animals were housed in positive-pressure air conditioned units (25°C, 50% relative humidity) and kept on a 12 h light/dark cycle. Water and mouse chow were provided *ad libitum.* All the experimental protocols were conducted strictly in compliance with the Guidelines notified by the Committee for the Purpose of Control and Supervision on Experiments on Animals (CPCSEA), Ministry of Environment and Forest, Government of India (CPCSEA guidelines, www.envfor.nic.in/divisions/awd/cpcsea_laboratory.pdf). The study was duly approved by JNU Institutional Animal Ethics Committee (IAEC Approved Project Code: 5/2010). All mice were randomly assigned to experimental groups. Experiments were designed so as to use the minimum number of mice. Numbers of mice used in various experiments are provided in figure legends. Mice were administered the required dose of paraquat by intra-peritoneal injections on alternate days. For the analysis of blood erythrocytes, 25 to 50 µl blood samples were taken weekly from tail-vein at different indicated time points as per prescribed CPCSEA guidelines. For deriving bone marrow and spleen cells, mice were euthanized by cervical dislocation before the organs were dissected out. All efforts were made to minimize pain.

### Reagents and other Supplies

Biotin-X-NHS (N-hydroxysuccinimide ester of biotin) was obtained from Calbiochem (La Jolla, CA). Streptavidin Allophycocyanin (SAv-APC), rat anti-mouse Ter-119-APC, rat anti-mouse Ter-119-FITC, anti-mouse CD16/CD32 were from BD Biosciences (San Diego, CA, USA). Anti-mouse CD71-PE, anti-mouse CD71-biotin, Annexin-V-FITC, anti-mouse IgG1κ-PE, anti-mouse IgG2bκ-APC, anti-mouse IgG2bκ-FITC, 7-Aminoactinomycin D (7-AAD) were procured from e-biosciences (San Diego, CA, USA). 5 (and 6) chloromethyl-2, 7 dichloro-dihydro-fluorescein diacetate (CM-H_2_DCFDA) was purchased from Molecular Probes (Eugene, OR, USA). Mouse Easy Sap biotin selection kit was procured from Stem cell technology (USA). Revert Aid First strand cDNA synthesis kit was from Thermo Scientific (India). Power SYBR green PCR Master mix was from Applied Biosystem (CA, USA). Fetal bovine serum was obtained from Hyclone (South Logan, UT). TRI reagent, RPMI, HEPES, Thiazole Orange (TO), Dimethylformamide (DMF), Paraquat and other analytical reagents were from Sigma-Aldrich (India).

### Double *In vivo* Biotinylation (DIB) Technique

Rationale and details of the DIB technique, its use in studying erythrocyte turnover and age related changes in erythrocytes *in vivo*, have been described elsewhere [18]–[24]. The DIB technique can be used for tracking changes in a cohort of erythrocyte that enters blood circulation within a defined 5 days window. Changes in relative proportions of the erythrocyte cohort within circulating erythrocytes and age related phenotypic changes from the time of its entrance of the cohort in blood till the end of its life span in circulation can be assessed by the DIB technique [24]. In the DIB technique the first step of high intensity *in vivo* biotinylation of all erythrocytes in blood is carried out by administration of biotin-X-NHS Ester (BXN) (three daily intravenous (i.v.) injections of 1 mg BXN dissolved in 20 µl of dimethyl formamide (DMF) and 250 µl of phosphate buffered saline). Mice are rested for 5 days and fresh erythrocytes that entered the blood after the first biotinylation step were biotinylated at a lower intensity by a single i.v injection of 0.6 mg of BXN dissolved in 12 µl of DMF and 250 µl of PBS. At any time point after the second biotinylation step, biotin intensity on circulating erythrocytes could be analyzed flow cytometrically after staining the cells with streptavidin coupled to an appropriate fluorochrome. Biotin^negative^ erythrocytes in circulation would represent fresh erythrocytes released in blood after the second biotinylation step. Biotin^l^°^w^ erythrocytes would represent the cohort of erythrocytes released in blood between the first and the second biotinylation steps, and biotin^high^ erythrocytes would represent the population of residual erythrocytes that were present in the blood at the time of first biotinylation step [24].

### Paraquat Treatment and Sample Collection

Paraquat dichloride hydrate (Sigma Chemicals, St. Louis, MO) was freshly dissolved in PBS for each injection. Mice were administered repeated doses of paraquat (10 mg/kg of body weight) intra-peritonealy on alternate days [8]. Control mice received vehicle alone. Blood samples (25–50 µl) were collected at different time points from tail vein in PBS containing 5 mM EDTA. Erythrocyte numbers and hemoglobin levels were estimated by using an electronic hematology particle counter (Melet Scholesing, MSE4 laboratories). Bone marrow (BM) cells were flushed out of femur and tibia using a 25-gauze needle and resuspended in RPMI medium with 10% FBS. Single cell suspensions of spleen cells were made by gently teasing the spleen in a small volume of PBS. Splenic and BM cells were strained through a fine sieve, pelleted by centrifugation, washed and suspended at desired concentration in RPMI medium with 10% FBS.

### Measurement of Intracellular Reactive Oxygen Species (ROS)

Intercellular ROS levels were assessed as described before [13]. Briefly, erythrocytes were washed and resuspended in pre-warmed PBS supplemented with 2% FBS and incubated with CM-H_2_DCFDA stain (5 µM) in the dark for 30 minutes at 37°C in an atmosphere of 5% CO_2_ in air. The oxidative conversion of CM-H_2_DCFDA to its fluorescent product by ROS was measured immediately by flow cytometry.

### Estimation of Relative Levels of SOD1 mRNA Expression in Reticulocytes

Reticulocytes within the erythrocyte population were identified by CD71 staining. Blood cells were stained with biotinylated anti-mouse CD71 monoclonal antibody (3 µg/ml) in PBS supplemented with 2%FBS for 20 min at 4°C, and CD71^+^ reticulocytes were sorted by using Easy Sap biotin cocktail and magnetic beads reagent according to manufacturer’s instruction (Stem cell technology) [25]. Purified reticulocyte preparations were about 90% pure (Ter-119^+^, CD71^+^ cells). Total RNA in sorted reticulocytes was extracted by TRI reagent (Sigma) and reverse transcribed into cDNA by utilizing the First Strand cDNA synthesis kit (Thermo Scientific, India) as per manufacturer’s instructions. Relative levels of SOD1 mRNA in reticulocytes were estimated by quantitative RT-PCR (Primers 5′-GAGACCTGGGC AATGTGACT-3′ and 5′-TTGTTTCTCATGGACCACCA-3′) as described before [13]. The RNA transcripts for SOD1 were quantified by SYBR green method by utilizing Real Time PCR. Expression levels of SOD1 mRNA were normalized to 5s rRNA levels using the 2^−ΔΔCT^ method [26].

### Flow Cytometric Analysis for Phenotype, Cell Cycling and Apoptosis

Mouse blood was collected in PBS containing 5 mM EDTA and washed 3 times with ice cold saline containing HEPES buffer (10 mM, pH-7.4) and 1% FBS. Biotin labeled erythrocytes were stained with streptavidin-FITC or streptavidin-APC and analyzed on a flow cytometer as described before [24].

In some experiments, reticulocyte population in blood was enumerated flow cytometrically as described before [27] by staining with thiazole orange (TO, 50 ng/ml for 30 min at room temperature) that stains the residual RNA present in reticulocytes after the enucleation of the erythroid precursor cells.

For enumerating erythroid cells at different stages of differentiation in bone marrow and spleen, freshly prepared single cell suspensions from bone marrow or spleen were first incubated with anti-CD16/32 antibody (Fc block, 1 µg/10^6^ cells in 50 µL of PBS+2%FBS) for 10 min followed by staining with anti-CD71-PE and anti Ter-119-APC for 20 minute at 4°C [12], [13]. After incubation, cells were washed and analyzed on a BD FACS Calibur flow cytometer using Cell Quest software for acquisition and analysis.

For cell cycle study, methodology of Marinkovic *et al.* [13] was used. Single cell suspensions of bone marrow and spleen cells were stained with Ter-119-FITC and Propidium iodide (PI) and PI staining pattern of Ter-119 gated cells was analyzed using Modfit-LT software that enumerated proportion of cells in G1/G0, S and G2M phases. For the analysis of apoptotic response, cells were stained with Annexin V-FITC and 7-AAD dye as described before [12]. Bone marrow and spleen cells were washed with PBS containing 2% FBS, resuspended in the PBS containing 2.5 mM calcium chloride and stained with Annexin V-FITC and 7-AAD along with Ter-119-APC antibody for 20 minutes at room temperature. Cells were washed and resuspended in the PBS with 2.5 mM calcium chloride (CaCl_2_) for immediate flow cytometric analysis.

### Statistical Analysis

Statistical analysis was carried out by using Sigma plot software. Standard t-tests were used to calculate the significance levels between groups. *p*<0.05 was considered significant.

## Results

### Paraquat Induced Anemia in Mice

**Figure 1:**
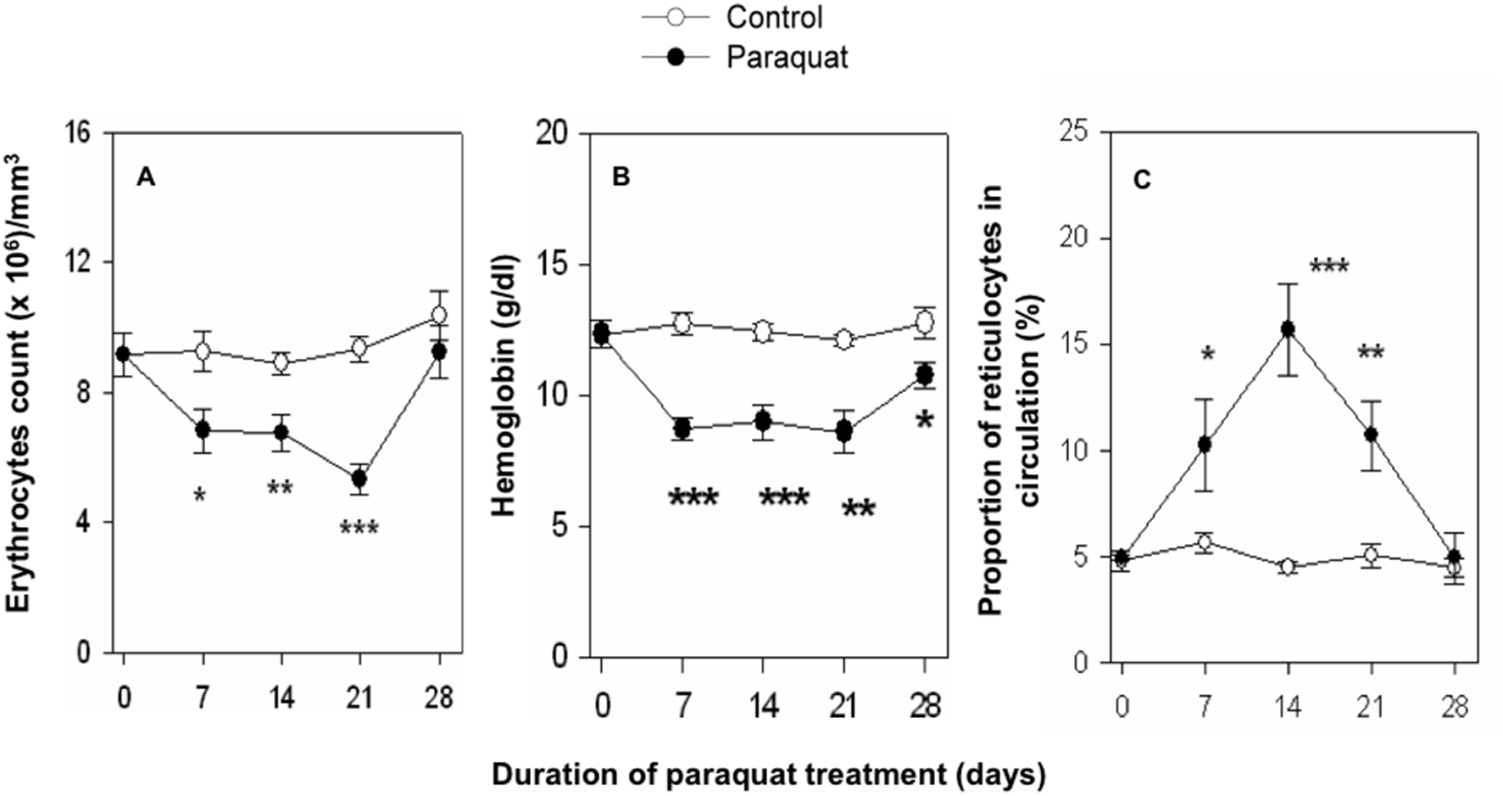
Induction of anemia and reticulocyte surge in paraquat treated mice. Mice were administered repeated doses (10 mg/kg body weight, i.p on alternate days) of paraquat and blood samples were collected at different time points. Blood erythrocyte count (panel A) and hemoglobin levels (panel B) were measured using an electronic hematology particle counter at different time intervals. Reticulocyte estimation (panel C) was done by thiazole orange (TO) staining and flow cytometric analysis. Data is represented as mean ± SEM; n = 6 in control and 6–8 in treated groups. *p<0.05, **p<0.005 and ***p<0.0005.

While the occurrence of anemia in subjects exposed to paraquat has been documented [9], [10], [28], the nature of paraquat induced changes in the turnover of blood erythrocytes and erythropoietic processes are not known. In order to study such changes, we developed a mouse model where repeated administration of paraquat (10 mg/kg of body weight i.p. on alternate days) induced a significant decline in blood erythrocyte count (range 26%–45% decline) as well as blood hemoglobin levels (range 25%–38% decline) on 7, 14 and 21 day time points (Figure 1A and B). Erythrocyte count as well as blood hemoglobin levels however returned to normal by the end of 4 week, even though the paraquat treatment was continued. Results in Figure 1C further show that there was a marked surge of reticulocyte count in blood circulation in paraquat treated mice. Maximum increase in reticulocyte count (about 4.5 fold) occurred on day 14 of paraquat treatment (Figure 1C). As the blood erythrocyte count normalized by day 28 of paraquat treatment, the reticulocyte percentage in blood also fell back to normal (Figure 1C). These results show that paraquat treatment induced a transient anemia in mice that is associated with a surge of reticulocytes representing enhanced erythropoietic activity.

### Turnover of Erythrocytes in Paraquat Treated Mice

**Figure 2:**
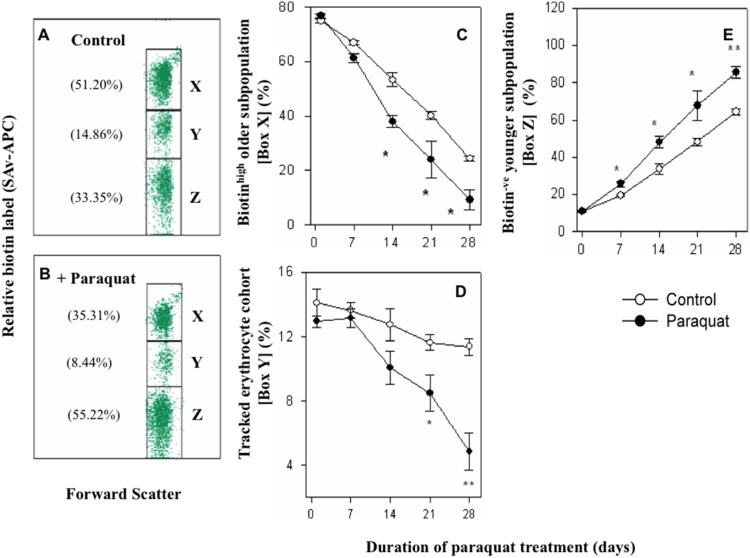
Effect of paraquat on erythrocyte turnover. Double biotinylation technique was used to label erythrocytes *in vivo* as described in methods. Mice were administered three daily i.v. doses of 1 mg biotin-X-NHS Ester (BXN) (first biotinylation step). After 5 days rest, a single i.v. dose of 0.6 mg BXN was administered (second biotinylation step). Repeated doses of paraquat (10 mg/kg) were administrated i.p. on alternate days starting the day after the second biotinylation step. Blood samples were collected on day 0 just before the first paraquat injection, and at 7, 14, 21 and 28 day time points during the paraquat administration. Erythrocytes in the blood samples were stained with streptavidin-APC and analyzed on flow cytometer. Box-Y in panel A and B contain erythrocytes that entered the circulation between the first and the second biotinylation step in control and paraquat treated mice respectively. Erythrocytes younger than those in box-Y are in box-Z (biotin^negative^ population) whereas the erythrocyte population older than the population in box-Y is represented as events in box-X (biotin^high^ population). Panel C shows the survival kinetics of older erythrocytes (box-X) in control and paraquat treated mice. Panel D shows the kinetics of survival of the tracked cohort of box-Y erythrocytes in control and paraquat treated mice. Panel E shows the kinetics of accumulation of younger box-Z erythrocytes in control and paraquat treated mice. Data is represented as mean ± SEM; n = 6 in control and 4–5 in treated groups. *p<0.05, **p<0.005 and ***p<0.0005.

In most types of anemia, it is not known whether age of erythrocytes in circulation has a bearing in the elimination of erythrocytes. Turnover of erythrocytes of different age groups in blood circulation in control and paraquat treated mice was studied by using a recently developed technique involving double *in vivo* biotinylation (DIB technique) of circulating erythrocytes (24). In this technique a cohort of erythrocytes entering blood circulation within a defined window of 5 days can be tracked as it ages in real time in blood circulation. This cohort is identified as erythrocyte subpopulation in box “Y” (biotin^l^°^w^ subpopulation of blood erythrocytes) in Figure 2A (control mice) and 2B (paraquat treated mice). Erythrocytes in box “X” (biotin^high^ subpopulation of erythrocytes) represent older erythrocytes of age greater than that of the biotin^l^°^w^ cohort of erythrocytes being tracked, and erythrocytes in box “Z” (biotin^negative^ subpopulation of erythrocytes) represent younger erythrocytes of age lower than the tracked cohort of erythrocytes (Figure 2A, B). Decline in biotin^l^°^w^ subpopulation from 14.86% in control to 8.44% (43% decline) in paraquat treated mice indicates that 14 days after the paraquat treatment, the cohort of erythrocytes that entered the blood immediately before paraquat treatment started, had declined 43% more as compared to the similar cohort in control mice (Figure 2).

Time kinetics of changes in subpopulations X, Y and Z in control and paraquat treated mice are shown in Figure 2 C, D and E respectively. Erythrocyte cohort entering blood circulation at zero time point was only moderately depleted till 28 day time point in control mice, but fell steeply in paraquat treated mice (Figure 2D). These results indicate that while the erythrocytes that entered blood circulation in control mice were relatively stable, the corresponding survival in paraquat treated mice was only about 30% at 28 day time point. Younger erythrocyte populations that are stable in control mice thus seem to undergo significant elimination in paraquat treated mice. The kinetics of changes in subpopulations X and Z indicate that the proportion of younger subpopulation of erythrocytes were significantly greater (Figure 2E) whereas the older subpopulation of erythrocytes (Figure 2C) was significantly reduced in blood circulation in paraquat treated mice. Even though the younger erythrocytes were eliminated at a higher rate in paraquat treated mice, increased proportion of younger erythrocytes in circulation of paraquat treated mice was due to a large surge of reticulocytes that that takes place in the anemic phase (Figure 1C). A decline in the relative proportion of older erythrocytes in paraquat treated mice could be a consequence of a large relative proportion of younger erythrocytes in the circulation or enhanced elimination of old erythrocytes.

### ROS Production in Erythrocytes from Paraquat Treated Mice

**Figure 3:**
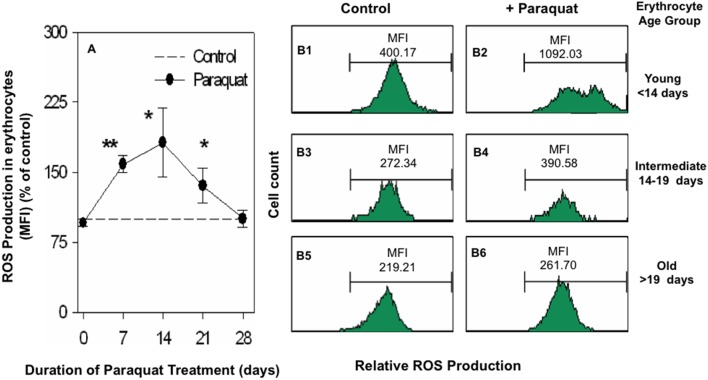
Levels of ROS in erythrocytes from control and paraquat treated mice. Mice were treated with paraquat (10 mg/kg i.p. on alternate days) for 28 days. At different given time points, blood samples were taken and ROS levels in erythrocytes was estimated flow cytometrically after treatment with CMH_2_DCFDA dye as described in methods. Summarized time kinetics data from 5 mice, depicting relative levels of ROS in erythrocytes in response to paraquat treatment are shown in panel A where control MFI due to ROS generation in control erythrocytes was taken as 100 and ROS levels in paraquat treated mice depicted in relative terms [*p<0.05 and **p<0.005]. Representative histograms showing the levels of ROS in erythrocytes of different age groups from control and paraquat treated mice are shown in panels B1 to B6. Mice were DIB labeled and treated with paraquat as described in legends to Figure 2. After 14 days of the paraquat treatment, blood cells were stained with streptavidin-APC to delineate the biotin^negative^, biotin^l^°^w^ and biotin^high^ subpopulations of erythrocytes. For ROS estimation, cells were incubated with CMH_2_DCFDA dye as described in methods. Fluorescence generated in biotin^negative^ subpopulation (younger subpoplation, panel B1, B2), biotin^l^°^w^ subpopulation (intermediate age subpopulation, panel B3, B4) and biotin^high^ subpopulation (older erythrocyte subpopulation, panel B5, B6) was estimated flow cytometrically.

ROS levels in erythrocytes were estimated by staining with CM-H_2_DCFDA, a dye that is converted to a fluorescent product by ROS. Paraquat treatment resulted in a significant increase in the level of ROS in the whole blood erythrocyte population. Maximum increase was noted at 14 day time point (Figure 3A). Since younger erythrocytes were eliminated at a higher rate in paraquat treated mice, it was important to correlate the elimination of erythrocytes with the cellular levels of ROS. DIB technique was used to demarcate circulating erythrocytes into different age groups as described above (Figure 2) and ROS levels were compared in erythrocytes of different age groups isolated from control and paraquat treated mice. Results in Figure 3B showing representative flow cytometric data of ROS levels indicate that maximum increase in cellular ROS levels occurred in the youngest erythrocytes (Figure 3, panels B1, B2) whereas no effect on ROS levels was observed in older group of erythrocytes (Figure 3, panels B5, B6). Increase in ROS levels was only marginal in erythrocytes of intermediate age group (Figure 3, panels B3, B4). Faster elimination of younger erythrocytes in paraquat treated mice is thus well correlated with increased ROS levels in this population of erythrocytes.

Superoxide dismutase (SOD) encoded in SOD1 gene, is an enzyme that deactivates superoxide radicals, which is an important component of ROS. Reduced SOD levels have been shown as a causative factor in anemia [29], [30]. Reticulocytes that constitute the youngest stage of erythrocytes lack nucleus but still continue to synthesize proteins by using residual mRNA that persists for some time after the enucleation process [31]. We studied the residual SOD1 mRNA levels in reticulocytes isolated from control and paraquat treated mice. Reticulocytes were isolated by using magnetic bead sorting, mRNA isolated and quantitative RT-PCR carried out after preparing the cDNA using reverse transcriptase as described in methods. The relative levels of SOD1 mRNA in reticulocytes from paraquat treated mice was 0.70±0.14 (n = 3, p = 0.02), taking the corresponding mRNA levels in control reticulocytes as 1. These results indicate that the levels of SOD1 mRNA in reticulocytes from paraquat treated mice were significantly lower than the control levels.

### Erythropoietic Activity in Paraquat Treated Mice

Bone marrow and spleen are two prime erythropoietic sites in adult mice [32], [33]. Erythroid cells in bone marrow and spleen express Ter119 and CD71 markers and based upon the levels of expression of these two markers, four distinct stages of erythroid differentiation have been identified [12]–[14]. These stages include early proerythroblast (Ter^med^ CD71^high^), early basophilic erythroblast (Ter^high^ CD71^high^) (erythroblast A), late basophilic, polychromatophilic erythroblast and orthochromatophilic erythroblast (Ter^high^ CD71^med^) (erythroblast B) and orthochromatophilic erythroblast with mature erythrocytes (Ter^high^ CD71^l^°^w^) (erythroblast C). Total erythroid population in bone marrow can be enumerated by setting an inverted L-shaped gate in flow cytometric histograms that include all the above mentioned four stages of erythroid differentiation [12]–[14].

**Figure 4:**
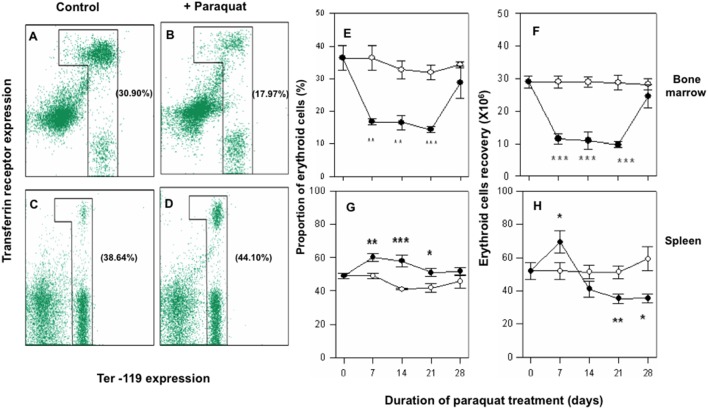
Gating of erythroid cells in bone marrow and spleen cell preparations and time dependent changes in relative proportions and absolute recoveries of erythroid cells in bone marrows and spleens of control and paraquat treated mice. Mice were treated with repeated doses of paraquat (10 mg/kg, i.p) on alternate days. Bone marrow and spleen cells were harvested just before the beginning of paraquat treatment and at different time points thereafter. Proportion of erythroid cells in bone marrow and spleen was determined by staining with anti-mouse Ter 119-APC and anti-mouse CD71-PE antibody and enumeration of cells falling within the inverted L-shaped gate as described in methods. Panels A, B, C and D show representative flow cytometric histograms depicting the inverted L shaped gates that defined the erythroid populations in bone marrow (panels A, B) and spleens (panels C, D) of control (left panels) and paraquat treated (right panels) mice. Values in parentheses next to the gated cells represent erythroid cells as percentage of all bone marrow (panel A, B) or spleen cells (panels C, D). Panels E, and F show summarized data of time dependent changes in the proportions (panel E) and absolute recoveries (panel F) of erythroid cells in bone marrow of control (○) and paraquat treated (•) mice. Similar data for spleen cells is shown in panels G and H). Data in panels E to H represents mean ± SEM; n = 6 in each groups. *p<0.05, **p<0.005 and ***p<0.0005.

To investigate the effect of paraquat treatment on erythropoiesis, we examined the relative proportions as well as absolute recoveries of erythroid cells in bone marrow and spleen of control and paraquat treated mice. Results of a representative experiment show that 30.9% of the cells in control bone marrow belonged to erythroid lineage. In paraquat treated mice, the proportion of erythroid cells fell to 17.97%, suggesting a significant fall in erythropoietic activity in bone marrow (Figure 4A and B). In contrast, the erythroid population in spleen expanded from 38.64% in control mice to 44.1% of spleen cells in paraquat treated mice (Figure 4C and D). Time kinetics data on changes in erythroid populations in bone marrow in paraquat treated mice show a steep fall in percentage of erythroid cells in bone marrow on day 7 (Figure 4E). That decline was sustained on day 14 and 21 time points and a recovery occurred thereafter (Figure 4E). Changes in absolute recovery of erythroid cells from the bone marrows of paraquat treated mice (Figure 4F) paralleled the changes observed in relative proportion of these cells.

In contrast to the observation in bone marrow, the proportion of erythroid cells increased significantly (range 21% to 41% increase) in spleens of paraquat treated mice especially on day 7, 14 and 21 day of treatment (Figure 4G). However the recovery of erythroid cells was significantly higher (up to 33%) only on 7 day time point and a significant decline was observed on 21 and 28 day time points (Figure 4H). These results suggest that treatment with paraquat resulted in a suppressed erythropoietic activity in bone marrow but an increased level of erythropoietic activity in spleen.

**Figure 5:**
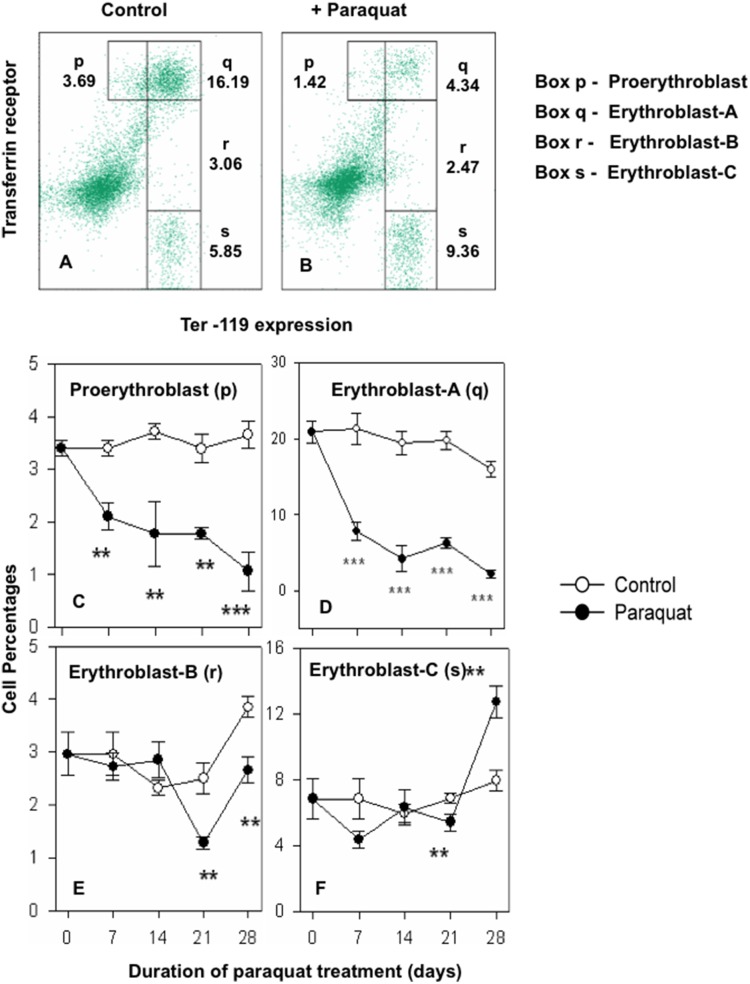
Time kinetics of paraquat induced changes in the proportions of cells in different stages of erythroid differentiation in bone marrow. Mice were treated with paraquat and bone marrow cells were harvested as described in legends of Figure 4. Bone marrow cells were stained with anti-mouse Ter 119-APC and anti-mouse CD71-PE antibodies followed by flow cytometric analysis to delineate four distinct stages (p, q, r and s) in erythroid differentiation as described in the text. Panels A and B illustrate typical gating of for the four stages (p, q, r and s) in bone marrows from control and paraquat treated mice respectively. Values in parentheses next to the gated cells represent the percentage values of different subpopulations of erythroid as percentage of all bone marrow cells. Panels C, D, E and F illustrate the kinetics of changes in proportions of proerythroblast (population-p), erythroblast A (population-q), erythroblast B (population-r) and erythroblast C (population s) respectively in bone marrows of control (○) and paraquat treated (•) mice. Data represents mean ± SEM; n = 6 in each groups. *p<0.05, **p<0.005 and ***p<0.0005.

**Figure 6:**
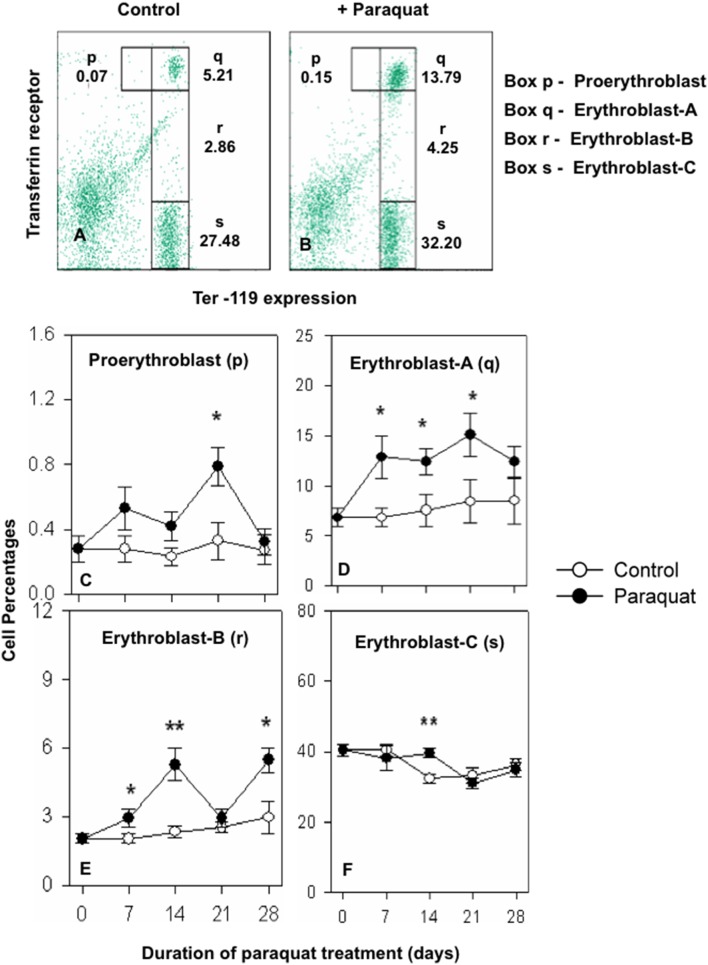
Time kinetics of paraquat induced changes in the proportions of cells in different stages of erythroid differentiation in spleen. Mice were treated with paraquat and spleen cells were harvested as described in legends of Figure 4. Spleen cells were stained with anti-mouse Ter 119-APC and anti-mouse CD71-PE antibodies followed by flow cytometric analysis to delineate four distinct stages (p, q, r and s) in erythroid differentiation as described in the text. Values in parentheses next to the gated cells represent the percentage values of different subpopulations of erythroid as percentage of all spleen cells Panels A and B illustrate typical gating of for the four stages (p, q, r and s) in spleen cells from control and paraquat treated mice respectively. Panels C, D, E and F illustrate the kinetics of changes in proportions of proerythroblast (population-p), erythroblast A (population-q), erythroblast B (population-r) and erythroblast C (population s) respectively in the spleens of control (○) and paraquat treated (•) mice. Data represents mean ± SEM; n = 6 in each groups. *p<0.05, **p<0.005. Values in parentheses represent percentage of erythroid cells.

Since there are four well defined stages of erythroid differentiation in bone marrow and spleen [12]–[14], it was important to find out whether the observed changes in erythropoietic activities were generalized or restricted to some specific erythroid subpopulations. Kinetics of paraquat induced alterations in relative proportions of the four discrete stages of erythroid differentiation were therefore examined in bone marrow and spleen. Figure 5A and B illustrate the gates used for the enumeration of the four stages of erythroid differentiation (p, q, r and s) in bone marrow. Figure 5C to 5F show kinetics of changes in the proportion of p, q, r and s subpopulations in control and paraquat treated mice. Two early stages of differentiation pro-erythroblast (p) and erythroblast-A (q) were significantly depressed (range 55 to 85% decline) after paraquat treatments but the effect was marginal in later two stages of differentiation i.e. erythroblast-B (r) and erythroblast-C (s) (Figure 5). Figure 6 shows similar data for alterations in the four erythroid subpopulations in spleens of control and paraquat treated mice. Figure 6A, B show the gates used for delineating the p, q, r and s stages of erythrocyte differentiation in spleen. Time dependent changes in the proportions of populations p, q, r, and s in control and paraquat treated mice is shown in Figure 6, panels C to F. In contrast to bone marrow, an enhancement was seen at all time-points in the relative proportions of proerythroblast (p), erythroblast-A (q) and erythroblast B (r) stages of erythroid differentiation in spleens of paraquat treated mice (Figure 6 C–F).

### Effect of Paraquat on Proliferative and Apoptotic Responses in Erythropoietic Cells

Changes seen above in the relative proportions of erythroid subpopulations in bone marrow and spleen could be due to changes in proliferative activity or cell death of the cells in erythroid lineage, or both. Cell cycle was analyzed in the erythroid population of bone marrow and spleen cell by staining the cells with Ter119 antibody and propidium iodide (PI). Cell cycle analysis was carried out on gated Ter119^+^ erythroid cell populations. Typical results of the cell cycle analysis are provided as supplementary data (Figure S1). Combined results of cell cycle data from 5 control and 5 paraquat treated mice are summarized in Table 1. Mean proportions of S and G2/M phase cells were 24.88% and 8.77% respectively in the bone marrow of control mice and these values fell to 17.62% and 2.67%, respectively, in paraquat treated mice. In spleen, S and G2/M cells comprised 3.01% and 3.05% erythroid cells in control mice but these proportions increased to 13.51% and 13.01% respectively in paraquat treated mice. These results indicate that while the proliferative activity declined in the erythroid cells in bone marrow, a significant increase of proliferative activity occurred in the erythroid cell population in spleens of paraquat treated mice.

**Table 1:** Changes in cell cycle pattern in bone marrow and spleen erythroid cells after paraquat treatment.

Organ	Treatment	Cell percentage		
		G1/G0	S Phase	G2M
Bone marrow	None	66.32±8.77	24.88±2.55	8.77±3.04
	Paraquat	78.31±2.40*	17.62±1.47*	2.67±0.73*
Spleen	None	93.94±3.93	3.01±2.32	3.05±1.63
	Paraquat	73.47±8.13*	13.51±3.11*	13.01±5.13*

Mice were treated with repeated doses (10 mg/kg) of paraquat on alternate days. Bone marrow/spleen cells were harvested after 14 days of exposure and stained with anti-mouse Ter 119-FITC and propidium iodide followed by flow cytometric analysis. Ter119^+ve^ cells were gated and cell cycle analysis was done on gated cells by utilizing Modfit-LT software. Each value represents mean ± SEM of data from 5–6 mice.

*p<0.05 Standard t test, comparisons of cells in different phases of cell cycle in control and paraquat treated mice.

Apoptotic and necrotic responses were also examined in Ter119^+^ erythroid cells in bone marrow and spleen in control and paraquat treated mice. Cells were stained with Ter119 antibody as well as with Annexin-V-FITC and 7-AAD. Typical results of the analysis of apoptotic cells is given as supplementary data (Figure S2). Combined data of apoptotic and necrotic responses from 5 control and 5 paraquat treated mice are summarized in Table 2. Significant accumulation of necrotic erythroid cells was observed in the bone marrow cells from paraquat treated mice. No significant effect of paraquat treatment was however observed in the levels of apoptotic or necrotic erythroid cells in spleens of paraquat treated mice.

**Table 2:** Paraquat induced apoptotic response in erythroid cells in bone marrow and spleen.

Organ	Treatment	Apoptotic cells *Annexin V +ve 7AAD −ve*	Necrotic cells *Annexin V +ve 7AAD +ve*
Bone marrow	None	21.80±3.43	1.79±0.49
	Paraquat	26.55±0.88	28.77±8.62*
Spleen	None	4.29±0.67	2.16±0.24
	Paraquat	4.69±1.21	2.63±0.85

Bone marrow/spleen cells were isolated from control and paraquat treated (10 mg/kg paraquat i.p. for 14 days) mice and stained with anti-mouse Ter 119-APC, anti-mouse CD71-PE antibody along with Annexin-V FITC and 7-AAD followed by flow cytometric analysis. Cells were gated on the basis of CD71 and Ter119 staining as shown in Fig. 6 and apoptotic cells were quantified in this gated population on the basis of Annexin V and 7-AAD staining. Each value represents mean ± SEM of data from 5–6 mice.

*p<0.05, Standard t test, comparisons of necrotic cells in control and paraquat treated mice.

Taken together, our results show that paraquat treatment depressed cell proliferative activity and enhanced cell death of erythroid lineage cells in bone marrow. In contrast, proliferation of erythroid cells was significantly enhanced in spleens of paraquat treated mice though the effect of cell death and apoptosis was marginal if any in spleen cells of paraquat treated mice.

## Discussion

While anemia as a consequence of exposure to paraquat has been documented [9], [10], [28] the mechanism for the same is not clear. In the present study we have studied the cellular and molecular events that occur in the erythroid system as a consequence of paraquat treatment. Intraperitoneal administration of paraquat (10 mg/kg body weight) on alternate days induced acute anemia in mice that peaked on day 14. Interestingly, however after 4 weeks of paraquat administration the blood erythrocyte count came back to normal. These results suggested that some kind of refractoriness to paraquat emerged in the erythroid system.

An important aim of this study was to find if the age of erythrocytes in circulation is a factor in determining their elimination in paraquat treated mice. We have recently developed a double *in vivo* biotinylation (DIB) technique that allows us to study erythrocyte cohorts of defined age groups in blood circulation [24]. Using DIB technique we have previously demonstrated that freshly released erythrocytes in blood are stable in blood till 12 days of age and are not eliminated from blood circulation within this time period [24]. From 12 to 40 days of age, erythrocytes are lost at an average rate of 1.3% per day and the rate of loss further increases to 2.8% from 40 to 60 days of age, by which time point virtually all erythrocytes of the cohort are lost from the circulation [24]. In the current study we found that the loss of erythrocytes in paraquat treated mice starts at a rate of above 2% per day right from the time these cells enter the blood (Figure 2D). These results clearly indicate that the resistance to elimination, a feature of young erythrocytes in control mice, is lost in paraquat treated mice indicating that young erythrocytes are randomly eliminated in paraquat treated mice. In a seemingly contradictory way, our results also show that the relative proportions of younger erythrocyte in blood actually increased in paraquat treated mice (Figure 2E). The increase is due to a marked compensatory surge of reticulocytes that occurs in paraquat treated mice (Figure 1C) since these reticulocytes are included in the biotin negative young cohort of erythrocytes. Within a day of entry into circulation, reticulocytes get converted into young erythrocytes and elimination commences in this population as shown in the survival curve (Figure 2D). A significant decline in the proportion of older erythrocytes is also seen in paraquat treated mice. This decline could merely be a reflection of enhanced proportion of younger cells in circulation or an increased susceptibility of old erythrocytes to elimination.

We found a significant increase in the levels of reactive oxygen species (ROS) in erythrocytes from paraquat treated mice. An increase in ROS levels coupled with the fact that erythrocytes lack nucleus and therefore robust self-repair mechanisms; suggest that increased ROS could be a contributory factor in the induction of anemia in paraquat treated mice. Further supportive evidence of an involvement of ROS in paraquat induced anemia is our finding of a significant decline in the mRNA levels for superoxide dismutase (SOD) enzyme in reticulocytes from paraquat treated mice. SOD deactivates superoxide radicals that constitute an important component of ROS and a decline in SOD expression in reticulocytes (that constitute a high proportion of new erythrocytes in paraquat treated mice, Figure 1) may render the erythrocytes more susceptible to ROS, resulting in loss of young erythrocytes. It should be pointed out that enhanced ROS production as well as decreased SOD1 mRNA levels may not be due to a direct effect of paraquat on reticulocytes. Modulatory effect of paraquat on these parameters may more likely be a hangover of similar changes in nucleated erythroid precursors in bone marrow and spleen that persist even after the enucleation of precursors and export of reticulocytes into blood. This proposition however needs further verification.

Using a standardized technique to delineate the cells of erythroid lineage by flow cytometric analysis of cells double stained with CD71 and Ter 119 monoclonal antibodies, we found that the proportion as well as the absolute numbers of cells of erythroid lineage declined sharply in the bone marrow of paraquat treated mice. This decline may be due to a toxic effect of paraquat as well as due to enhanced release of reticulocytes to compensate for the declined erythrocyte count in blood. Interestingly, a parallel increase was seen in the proportion of cells of erythroid lineage in the spleen of paraquat treated mice that could suggest a shift or extension of erythropoietic activity to spleen. It is possible that the enhanced erythropoietic activity in spleen may occur due to increase demand of fresh erythrocytes in circulation.

Paraquat treatment results in a severe and sustained depletion of pro-erythroblast and erythroblast A sub-populations in bone marrow (Figure 5). Corresponding results from spleen show that the two early stages of erythroid differentiation are significantly augmented in this organ. These results support the suggestion of the shift/expansion of erythropoietic activity to spleen in paraquat treated mice. Further support of the proposition is derived from our results that show a significant decline in the proliferative activity in erythroid population of cells in bone marrow and a marked boost in cell division activity in the cells of erythroid lineage in spleen. A substantial accumulation of necrotic erythroid cells was observed in bone marrow of paraquat treated mice that may denote the toxic effect of paraquat. Lack of presence of necrotic or apoptotic erythroid cells in spleen may be due to presence of a more active scavenging mechanism in spleen or lack of a strong toxic effect of paraquat in the spleen milieu.

The reason behind the transitory nature of anemia in paraquat treated mice is not known. We may speculate that mechanisms that oppose the cell damaging factors like the generation of ROS may eventually get up-regulated in paraquat treated mice. Work is currently underway in our laboratory to examine this proposition.

## Supporting Information

Figure S1 **Effect of paraquat treatment on cell cycling patterns in erythroid population of bone marrow and spleen.** Mice were treated with repeated doses of paraquat. Bone marrow and spleen cells were harvested after 14 days of treatment and stained with anti-mouse Ter 119-FITC and propidium iodide followed by flow cytometric analysis. Cell cycle analysis was done on Ter 119 gated cells by utilizing Modfit-LT software. Representative cell cycle data is shown for bone marrow cells derived from control (panel A) and paraquat treated (panel B) mice. Similar data for spleen cells is shown in panels C and D. Percentage values for cells in Go//G1, S and G2/M phases are given.
(TIF)

Figure S2 **Paraquat induced apoptotic and necrotic changes in bone marrow and spleen erythroid cells.** Mice were treated with repeated doses of paraquat. Bone marrow and spleen cells were harvested after 14 days of treatment and stained with anti-mouse Ter 119-APC, along with Annexin-V FITC and 7-AAD followed by flow cytometric analysis. Apoptotic cells were quantified in Ter119 gated population on the basis of Annexin V and 7-AAD staining. Panels A and B show the representative flow cytometric histograms for bone marrow cells derived from control and paraquat treated mice. Similar data for spleen cells is shown in panels C and D. Values in different quadrants represent percentage of cells. Apoptotic cells are in top right quadrants and the necrotic cells in lower right quadrants.
(TIF)
