# Supplementary figures and images for: Elimination of Young Erythrocytes from Blood Circulation and Altered Erythropoietic Patterns during Paraquat Induced Anemic Phase in Mice
Source: PLoS One. 2014 Jun 19;9(6):e99364. doi: 10.1371/journal.pone.0099364 (PMC4063733; doi:10.1371/journal.pone.0099364)

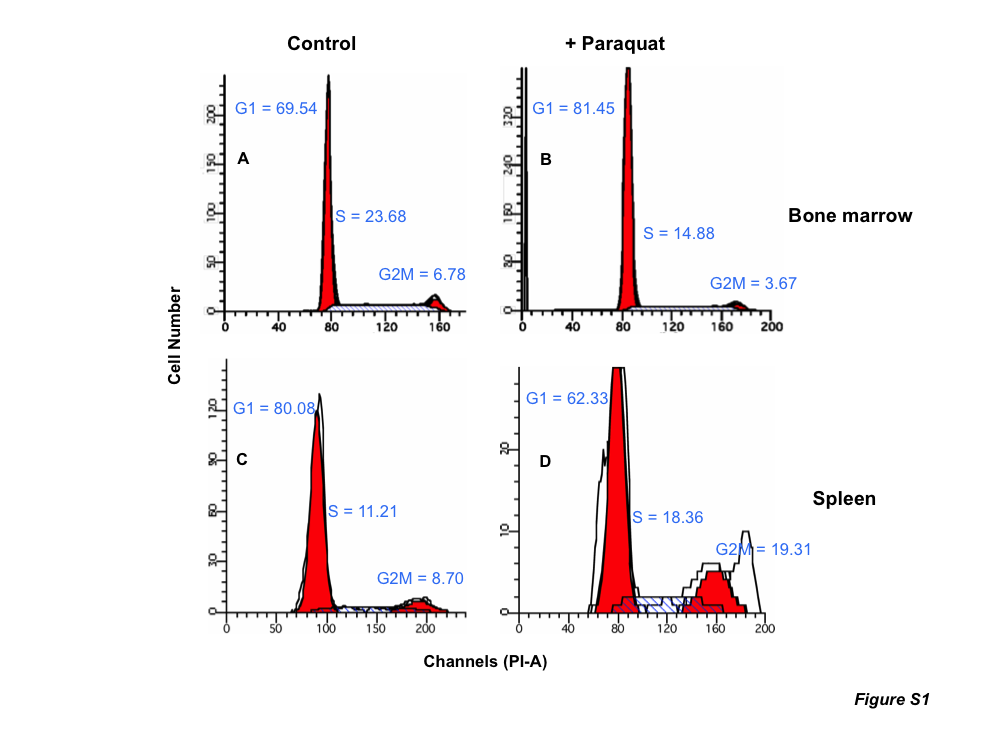

Supplement: Figure S1 — Effect of paraquat treatment on cell cycling patterns in erythroid population of bone marrow and spleen. Mice were treated with repeated doses of paraquat. Bone marrow and spleen cells were harvested after 14 days of treatment and stained with anti-mouse Ter 119-FITC and propidium iodide followed by flow cytometric analysis. Cell cycle analysis was done on Ter 119 gated cells by utilizing Modfit-LT software. Representative cell cycle data is shown for bone marrow cells derived from control (panel A) and paraquat treated (panel B) mice. Similar data for spleen cells is shown in panels C and D. Percentage values for cells in Go//G1, S and G2/M phases are given. (TIF) [file pone.0099364.s001.tif]

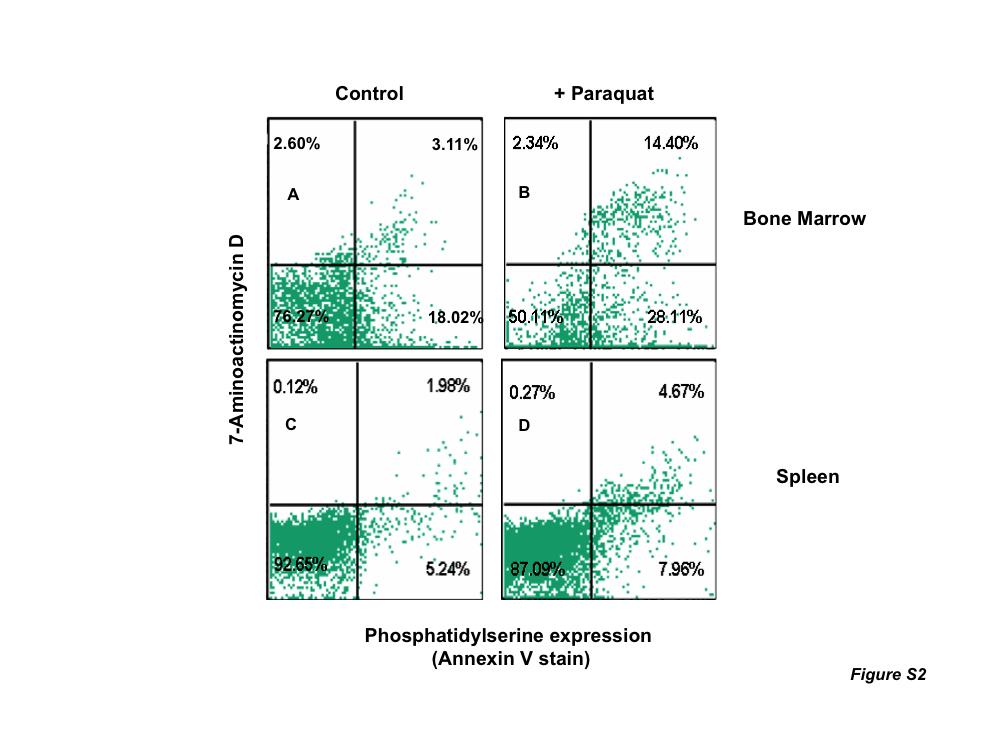

Supplement: Figure S2 — Paraquat induced apoptotic and necrotic changes in bone marrow and spleen erythroid cells. Mice were treated with repeated doses of paraquat. Bone marrow and spleen cells were harvested after 14 days of treatment and stained with anti-mouse Ter 119-APC, along with Annexin-V FITC and 7-AAD followed by flow cytometric analysis. Apoptotic cells were quantified in Ter119 gated population on the basis of Annexin V and 7-AAD staining. Panels A and B show the representative flow cytometric histograms for bone marrow cells derived from control and paraquat treated mice. Similar data for spleen cells is shown in panels C and D. Values in different quadrants represent percentage of cells. Apoptotic cells are in top right quadrants and the necrotic cells in lower right quadrants. (TIF) [file pone.0099364.s002.tif]
